# Supplementary material for: Antineutrophil Cytoplasmic Antibody-Associated Vasculitis and the Risk of Developing Incidental Tuberculosis: A Population-Based Cohort Study
Source: Medicina (Kaunas). 2023 Oct 30;59(11):1920. doi: 10.3390/medicina59111920 (PMC10673265; doi:10.3390/medicina59111920)
Supplement: Supplementary file 1 [file medicina-59-01920-s001.zip › medicina-2676709-supplementary.pdf]

Supplementary Table 1. Annual AAV incidence rate during study period.

Year	AAV<br>No. of events	Total Population<br>person years	Annual Incidence Rate per 100,000 person years
2000	336	937,649	35.83
2001	236	937,480	25.17
2002	226	926,038	24.41
2003	194	916,788	21.16
2004	215	909,605	23.64
2005	182	903,243	20.15
2006	195	897,042	21.74
2007	168	890,675	18.86
2008	143	884,089	16.17
2009	130	877,402	14.82
2010	121	870,735	13.90
2011	154	863,587	17.83
2012	119	855,691	13.91
